# Mutant calreticulin—Cancer driver and Achilles' heel for ET?

**DOI:** 10.1002/hem3.70368

**Published:** 2026-04-23

**Authors:** Moritz Reichert, Bethan Psaila

**Affiliations:** ^1^ MRC Weatherall Institute of Molecular Medicine and Ludwig Institute for Cancer Research University of Oxford Oxford UK; ^2^ Oxford University Hospitals NHS Trust Oxford UK

Essential thrombocythemia (ET) is a chronic blood cancer (Myeloproliferative Neoplasm, MPN) that arises following the acquisition of a mutation in a blood stem cell, causing enhanced signaling via the thrombopoietin receptor (TPO‐R). Dr. William Dameshek was the first to speculate that ET, polycythemia vera (PV), and myelofibrosis were part of a unifying pathology, remarking in an editorial in 1951 that they were “perhaps due to a hithero undiscovered stimulus.”^1^ Half a century later, four teams published that gain‐of‐function mutations in the cytokine signal transducer JAK2 (JA2V617F) were present in almost all PV and 2/3 of ET and myelofibrosis cases.^2^, ^3^, ^4^, ^5^ JAK inhibitors rapidly progressed through clinical development, and the first—ruxolitinib—was licensed for the treatment of myelofibrosis only 6 years after the identification of JAK2V617F. One year later, MPLW515L/K mutations were identified in 5% of patients with ET and myelofibrosis,^6^, ^7^ but the initiating driver mutation in the remaining 30% of JAK2/MPL mutation‐negative cases remained enigmatic. In 2013, two studies published by independent teams in the same issue of the *New England Journal of Medicine* identified mutations in calreticulin (mutCALR) in 70%–80% of these patients, identifying the second most frequent MPN cancer driver mutation.^8^, ^9^

## MUTANT CALRETICULIN (CALR)—THE UNEXPECTED CANCER DRIVER

A role for CALR in MPN pathogenesis came as a surprise, given that it was known as an endoplasmic reticulum (ER)‐resident chaperone protein and had not previously been implicated in cancer biology. The original publications described the most frequent mutations as a 52‐base pair (bp) deletion (Type 1) or a 5‐bp insertion (Type 2), but found that ~1 in 6 mutations were noncanonical. Crucially, all pathogenic mutations resulted in the same novel C‐terminal epitope,^8^, ^9^ creating an aberrant, positively charged neoepitope with deletion of the ER‐retention signal, “freeing” mutant CALR (mutCALR) from its normal intracellular localization.

**Figure 1:**
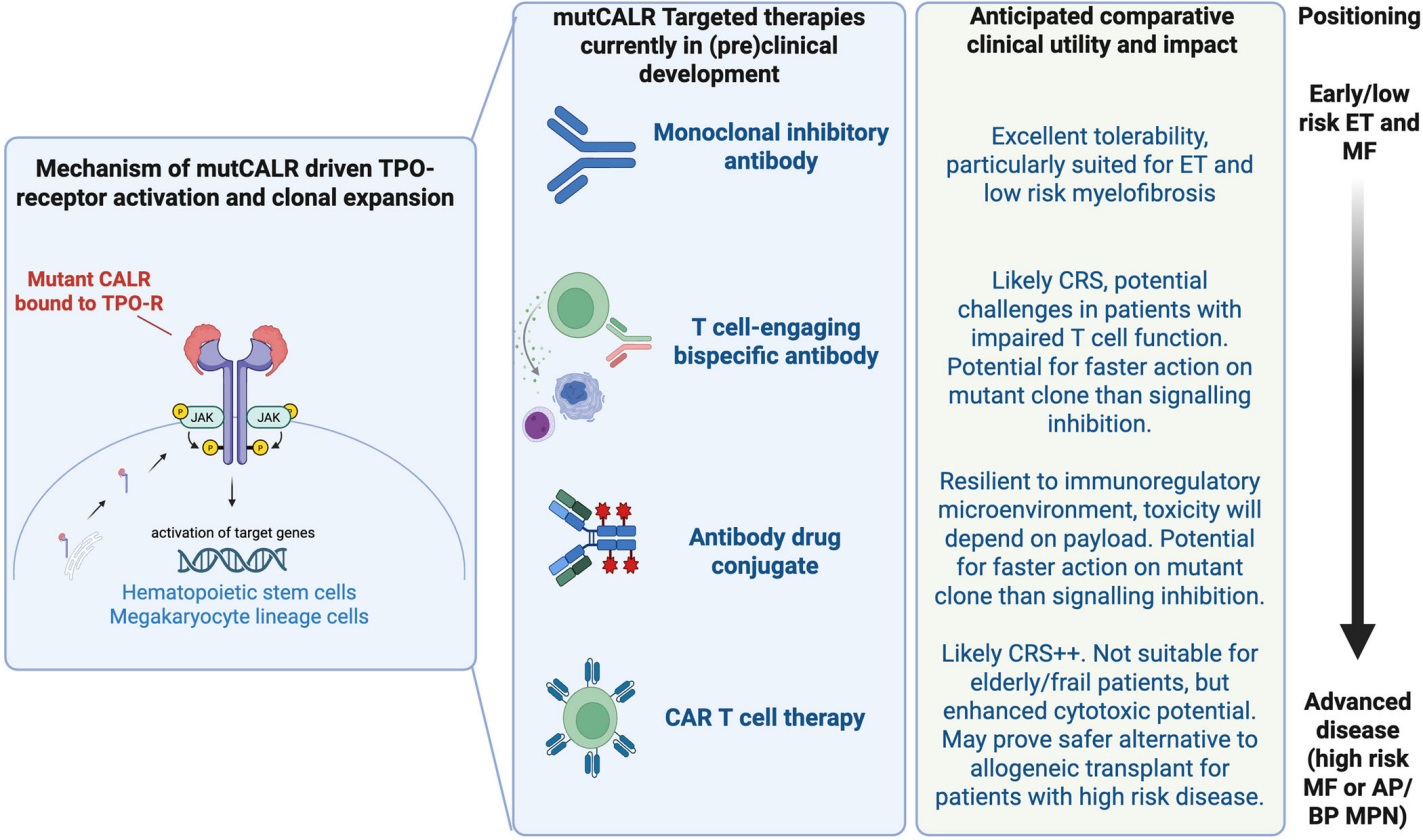
**Mutant CALR—Cancer driver and therapeutic target**. Left panel: schematic showing the mechanism by which mutant CALR (mutCALR) protein binds to the thrombopoietin receptor (TPO‐R) in the endoplasmic reticulum, then travels to the cell surface of TPO‐R‐expressing cells, inducing receptor dimerization, signaling activation, and altered gene expression, leading to a clonal advantage for TPO‐R‐bearing mutCALR+ hematopoietic stem cells and megakaryocyte‐lineage cells. Right panel: mutCALR targeting agents in clinical and pre‐clinical development. CRS, cytokine release syndrome; ET, essential thrombocytosis; TPO‐R, thrombopoietin receptor. Figure created with bioRender.com.

Subsequently, a series of publications confirmed the fascinating mechanism by which mutCALR triggers excess megakaryopoiesis, thrombocytosis, and myelofibrosis. First, the essentiality of the thrombopoietin receptor (TPO‐R) was demonstrated.^10^ This was followed by a description of the sequence of events in which the positively charged neoepitope forms an aberrant complex with the TPO‐R, and migration of the mutCALR‐TPO‐R complex to the cell membrane, triggering cytokine‐independent receptor activation and downstream signaling (Figure 1).

Fortuitously, from a therapeutic perspective, this also leads to a unique phenomenon in cancer biology: the direct product of a cancer‐driving oncogene being displayed as a cell surface neoantigen, creating an opportunity for molecularly targeted, cancer cell‐specific therapies. Notably, TPO‐R expression is restricted to hematopoietic stem cells (HSCs), megakaryocytes, platelets, and their intermediate progenitors—and since mutCALR requires TPO‐R for surface expression, the mutant protein is confined to these cells. As HSCs are the cancer‐initiating cells and megakaryocytes are the key drivers of fibrosis, mutCALR‐directed immunotherapies therefore primarily hit the cells responsible for both disease initiation and progression to advanced‐phase MPN.

## MUTCALR ET: LOW RISK, BUT NOT NO RISK

ET results in a significant burden of morbidity and mortality. The incidence of mutCALR+ MPNs is estimated at 1–2.5/100,000 population per year, with a prevalence of 9–24/100,000 individuals.^11^ Over 50% of patients with ET experience fatigue or vasomotor symptoms such as headache, light‐headedness, acral paresthesia, and erythromelalgia.^12^ Complications include thrombosis, bleeding events, progression to myelofibrosis, progression to leukemia, pregnancy complications, and immune compromise.

**Figure 2:**
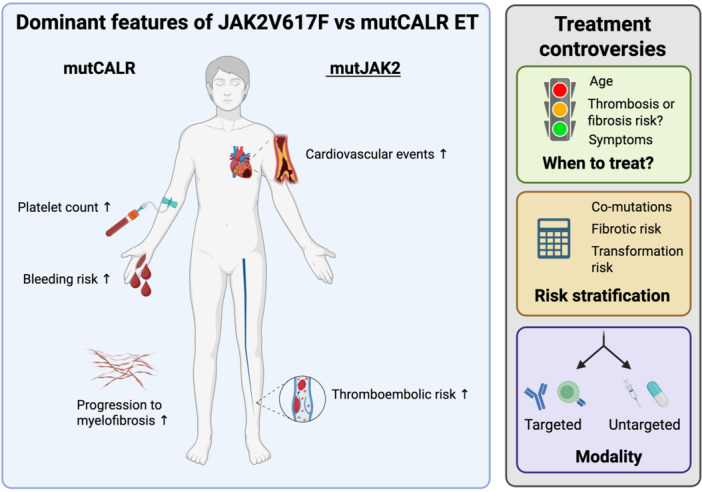
**Clinical differences and treatment controversies in patients with mutCALR versus mutJAK2 essential thrombocythemia**. Left panel: Patients with mutCALR ET demonstrate higher platelet counts, increased bleeding risk, and greater risk of progression to myelofibrosis compared to mutJAK2 ET patients. Conversely, mutJAK2 ET patients face increased risk of cardiovascular and thromboembolic events. Both groups experience common symptoms, including headaches, dizziness, visual disturbances, erythromelalgia, and fatigue, along with concerns about progression to myelofibrosis and secondary acute myeloid leukemia (AML). Right panel: Emerging clinical data and novel therapeutic modalities are beginning to raise questions regarding treatment indications, optimal risk stratification strategies, and selection of sequential treatment approaches. Figure created with bioRender.com.

Clear differences exist in the age of presentation and average platelet counts between JAK2V617F+ ET and mutCALR+ ET (Figure 2), with mutCALR+ cases being detected at a younger age (54 vs. 61 years for mutCALR vs. JAK2V617F+ ET) and with higher platelet counts (866 vs. 726 × 10^9^/L).^13^ Notably, although JAK2, MPL, and CALR mutations all cause thrombocytosis via pathological activation of TPO‐R signaling, their impact on platelet count and function appears distinct. In a review of over 1580 patients with MPN in the United States and Italy, the incidence of a platelet count >1000 × 10^9^/L was 53% for Type 1 mutCALR+ ET and 71% for Type 2 mutCALR+ ET, compared to only 34% for JAK2V617F+ ET.^14^ Despite the higher platelet counts, the cumulative incidence of thrombosis is lower for mutCALR+ ET—at around 10% at 10 years—compared to 20% for JAK2V617F+ ET,^8^ an observation that is accounted for in the IPSET Thrombosis Risk Score by incorporating the presence or absence of the JAK2V617F driver mutation in the risk prediction algorithm.^15^ The combination of more extreme thrombosis with reduced thrombotic risk for mutCALR ET as compared to JAK2V617F+ ET likely reflects a negative impact of the mutation on platelet function (reduced adhesion, spreading, and response to agonists),^16^ as well as an increased likelihood of acquired von Willebrand disease with extreme thrombocytosis.

## LESS THROMBOSIS, BUT MORE FIBROSIS

Patients with Type 1 mutCALR+ ET have an overall better survival but face a paradoxically higher incidence of bone marrow fibrosis compared to those with Type 2 mutCALR or JAK2V617F+ ET.^17^ This is somewhat counterintuitive, given that leukemic transformation is the leading cause of death in MPN patients^18^ and that evolution to leukemia is substantially more common from myelofibrosis than from ET or PV. Therefore, while progression to myelofibrosis is a definite concern with mutCALR ET, the overall impact of this on survival and clinical outcomes remains unclear.

The differences in clinical phenotypes between Type 1‐ and Type 2‐mutCALR‐driven MPNs are not completely understood but may relate to differences in loss of the calcium‐binding function with Type 1 but not Type 2 mutations,^19^ as well as their impact on downstream signaling pathways. In a recent study, the mutations were shown to differentially activate unfolded protein response (UPR) pathways, with type 1 mutant cells being more dependent on BCL‐xL for survival, potentially promoting TPO‐R signaling‐independent mutant cell survival.^20^ Although further research is required, it is possible that these features differentially impact megakaryocyte function and their capacity to drive fibrosis.

## MANAGEMENT OF MUTCALR+ ET—SHOULD THE FOCUS BE THROMBOSIS?

In current management algorithms for ET, the decision to initiate therapy is largely based on the risk of thrombosis, rather than disease progression.

### Antiplatelet therapy

The benefit of aspirin for primary prevention of thrombosis appears less certain in mutCALR+ ET than JAK2V617F+ MPNs, given the lower incidence of thrombosis and higher rates of extreme thrombocytosis and bleeding, and it may even confer a higher risk of bleeding without reducing thrombotic events.^21^ Consensus‐based recommendations from the European LeukemiaNet suggest low‐dose aspirin (75–100 mg/day) for patients with classical low‐risk ET who have concomitant cardiovascular risk factors, but endorse observation only (without antiplatelet therapy) in patients without cardiovascular risk factors, and advise using antiplatelet agents with caution if platelet counts are >1000–1500 × 10^9^/L due to the risk of bleeding.^22^

### Indications for cytoreduction, differential responses, and targets for therapy

Indications for cytoreduction are also less clear‐cut for mutCALR+ ET than for JAK2V617F+ MPNs. Non‐controversial indications include a prior history of thrombosis and/or platelet counts over 1500 × 10^9^/L, and those over the age of 60 who have significant cardiovascular risk factors.^22^ Whether patients over the age of 60 *without* cardiovascular risk factors also benefit from cytoreduction, and whether it is necessary to target a platelet count of < 400 × 10^9^/L, remains unclear. Further studies are required to clarify the benefits and intensity of cytoreduction in these groups.

Across ET and PV, first‐line cytoreductive therapies have not changed for several decades, with hydroxycarbamide generally used in older patients and interferon preferred in younger patients due to better tolerability and the added potential for disease modification—that is, molecular responses and possible prevention/resolution of fibrosis.^23^, ^24^, ^25^ However, just as for the indications to treat, responses to therapy also differ between mutCALR and JAK2V617F+ ET. Patients with mutCALR+ ET are less likely to benefit from a molecular response to interferon therapy, less likely to achieve complete hematologic responses to ruxolitinib, and the incidence of treatment‐emergent mutations appears higher.^26^ Similarly, subgroup analyses of patients with myelofibrosis receiving ruxolitinib therapy indicated that patients with mutCALR+ disease have more severe disease at baseline (in terms of anemia, % circulating blasts, and degree of fibrosis) and inferior spleen and symptom responses compared to patients with JAK2V617F‐mutated myelofibrosis.^27^ These data highlight the need for novel therapies for patients with mutCALR+ MPNs.

## FROM DRIVER TO ACHILLES' HEEL: A NEW ERA FOR MYELOPROLIFERATIVE NEOPLASMS

Given that current MPN therapies are not selective for the mutant clone, there is a huge interest in exploiting the opportunity for targeted therapy provided by the mutCALR neoantigen. Over the last 12–18 months, three pivotal clinical trials of mutCALR targeting therapies were initiated, including: a cancer vaccine administered with a checkpoint inhibitor (NCT05444530), a monoclonal inhibitory antibody (NCT05936359 and NCT06034002), and a T cell‐engaging bispecific antibody (NCT06150157), with trials of other agents anticipated in the next 1–2 years.

### Cancer vaccine targeting MPN driver mutations

Prior studies have shown that mutCALR epitopes are presented by MHC and can elicit T cell responses, but have also highlighted that chronic antigen exposure leads to T cell exhaustion.^28^, ^29^ A recent study of an adenoviral‐based vector vaccine (VAC85135, NCT05444530) containing polypeptides from both the mutCALR neoepitope and JAK2V617F administered in combination with the anti‐CTLA4 checkpoint inhibitor ipilimumab was terminated early this year due to disappointing results. While patients mounted sustained immunological responses to the adenovirus‐component of the vaccine, T cell responses to mutCALR were low and transient, and associated with no observable clinical benefit.^30^

### Monoclonal antibody therapy: INCA033989

Promising preliminary data was recently presented for the first‐in‐class monoclonal antagonist antibody INCA033989, a therapeutic that selectively targets mutCALR in complex with the TPO‐R, shown in preclinical studies to inhibit oncogenic signaling, leading to selective cytotoxicity of mutCALR+ cells.^31^ Two, phase 1/2 clinical trials (NCT05936359 and NCT06034002) began recruitment in late 2023 and are ongoing, enrolling patients with high‐risk ET refractory to at least one first‐line cytoreductive therapy and intermediate/high risk myelofibrosis, initially those who had previously been treated with or were ineligible for JAK inhibitor therapy. Early results shared at the European and American Society of Hematology 2025 Congresses for monotherapy in ET and myelofibrosis, and in combination with ruxolitinib for patients with myelofibrosis, reported impressive safety and efficacy signals. No dose‐limiting toxicities were observed, and over 90% of patients remained on study after 12 months. If this excellent tolerability is confirmed in future studies and with longer‐term follow‐up, this agent will be particularly attractive for patients with ET and low‐risk myelofibrosis (Figure 1). Rapid and durable normalization of the platelet counts was observed in the ET cohort—notable after only 2 doses, and 2/3 of patients were able to stop concomitant cytoreductive therapy, maintaining a normal or near‐normal platelet count (down from a median platelet count of >1000 × 10^9^/L). Durable hematological responses were evident at lower doses in patients with Type I CALR mutations than those with non‐Type 1 mutations. Most strikingly, nearly all patients (96%) achieved a reduction in mutCALR variant allele frequency (VAF), with 52% achieving reductions >25% and 31% achieving reductions >50%. Molecular responses were common alongside hematological responses.^32^ Encouraging efficacy was reported in the myelofibrosis cohort, with improvements in splenomegaly, symptoms, and anemia associated with molecular responses, although the tempo of clone size reduction appears slower in myelofibrosis than ET.^33^ The reasons for the slower molecular responses are unclear, and may relate to the greater burden of disease in myelofibrosis and higher abundance of mutCALR+ cell types that do not express the mutCALR‐TPO‐R on the cell surface, therefore are not directly vulnerable to mutCALR targeting therapies. Such cells would be expected to gradually reduce with time, if mutant HSCs are effectively targeted allowing healthy wildtype hematopoiesis to recover. Early translational analyses indicated clearance of mutant clone stem cells, megakaryocytes, and aberrant erythroid cells with normalization of bone marrow histology (reduced fibrosis and megakaryocyte clustering, increased proportion of non‐mutCALR+ megakaryocytes, and increased bone marrow erythropoiesis).^34^

### T cell‐engaging Bispecific Antibodies (BiTEs): JNJ‐88549968

INCA033989 is Fc‐silenced and therefore presumed to act independently of immune cell function. In contrast, JNJ‐88549968 is a bispecific, T cell‐engaging antibody (BiTE) that contains binding domains for both mutCALR and CD3, redirecting endogenous T cells to eliminate mutCALR+ cells.^35^ A phase 1 study is ongoing, in which a comparable safety profile to other T cell engagers would be predicted, with cytokine release syndrome expected to be among the therapy‐emergent adverse events, reflecting on‐target T cell activation. Given the encouraging data with INCA033989, this agent may also prove disease modifying.

### Antibody‐drug conjugates (ADCs)

Other modalities at earlier stages of development include mutCALR‐directed ADCs. Preclinical data were presented at the EHA and ASH 2025 congresses for ADCs with novel payloads, including SMARCA2/4 or CDK9 degraders.^36^ ADCs offer a compelling mechanism of action with targeted delivery of payloads directly to mutCALR‐expressing cells, combining the specificity of antibody‐targeting with cytotoxic potency of the payload, potentially offering more potent cytotoxicity than can be achieved with signaling inhibition or T‐cell engagement in the context of T cell exhaustion. Internalization is required for ADCs to exert their mechanism of action, and payload‐related toxicities will need to be considered.

### Chimeric antigen receptor‐expressing T cell (CAR‐T) cells: The ultimate weapon?

CAR T therapies targeting mutCALR are also in development,^37^, ^38^, ^39^ with preclinical evidence of selective cytotoxicity against patient cells in preclinical models, including human organoids and in vivo.^37^ The versatility and potency of CAR‐T cell therapies are appealing, as T cells can be engineered not only to express novel receptors (overcoming the necessity of target peptide‐HLA‐expression for T cell engagement) but also to secrete payloads that might combat a challenging tumor microenvironment (TME). Effective clearance of target cells and long‐lasting remissions have been demonstrated in other indications, but manufacturing costs and toxicities present ongoing challenges for CAR‐T cell therapeutics.

Overall, cell surface expression of mutCALR is intrinsically linked to MPL expression, and therefore varies across hematopoietic compartments, with higher levels on megakaryocyte lineage cells than HSCs. The low‐level expression on cancer‐initiating HSCs may hinder immunotherapies from achieving lasting molecular responses. However, CAR T cells can lyse target‐antigen‐expressing cells at antigen densities approximately 1000‐fold lower than those required for antibody‐mediated complement‐dependent cytotoxicity. It is conceivable that a CAR‐T cell therapeutic may enable superior targeting of mutant HSCs compared to a BiTE (albeit with increased cost and toxicities), although no direct comparisons have been published to date.

### Overcoming mutation‐type selectivity

CALR mutations, especially atypical variants, can result in structurally distinct C‐terminal neoepitopes that have a differential impact on signaling. These differences may impact the binding and efficacy of therapeutic antibodies directed against the mutant C‐terminus. In addition, increased TPO‐R dimerization, implying greater signaling activation, has been reported for Type 2 (ins5) versus other variants, which may impede the impact of inhibitory antibodies, at least at lower doses.^40^ Achieving potent targeting of Type 2 variants is particularly important, given that patients with Type 2 CALRmut ET have higher platelet counts (albeit a lower incidence of fibrosis), and those with myelofibrosis have overall worse outcomes than those with Type 1 variants. Mutation selectivity may vary between antibodies, depending on the specific epitope targeted. To further overcome mutation‐type selectivity, several distinct approaches have been proposed: **(1) Targeting the conserved N‐domain:** INCA035784 is a T‐cell redirecting antibody directed to the N‐domain, a region that is invariant across known CALR mutations and also conserved with wildtype CALR, but only exposed for binding when the protein is complexed with the TPO‐R. While no clinical data is available yet for this agent, targeting common regions of the C‐terminus or the N‐domain may be one way to overcome mutation‐type selectivity^41^; **(2) Combining antibodies that target distinct epitopes on the C‐terminus:** A second approach proposed to enhance antibody‐mediated signaling inhibition is to combine antibodies targeting distinct domains on the C‐terminus. A recent report combining proximal‐ and distal‐epitope binding antibodies demonstrated significantly enhanced signaling inhibition for Type 1 and Type 2 variants. While antibody combinations alone did not achieve complete inhibition for Type 2 CALR mutations, efficacy further synergy was demonstrated by combining dual antibody targeting with ruxolitinib, suggesting that patients with Type 2 variants may benefit from triple therapy with dual antibody targeting and JAK inhibition.^40^ **(3) Exploiting differential impact on signaling:** Differential signaling between Type 1 and Type 2 mutations are a potential therapeutic vulnerability, with differential dependence on BCL‐2 versus BCL‐xL targeting that might inform selection of targeted inhibitors.^20^

## UNRESOLVED QUESTIONS AND POTENTIAL CHALLENGES

The depth and rapidity of response reported for INCA033989 is unprecedented—raising hopes that mutCALR targeting therapies may represent the first truly disease‐modifying MPN therapies.^42^ However, despite the promise, key questions remain unresolved.

### Extinguishing the flames or turning down the gas?

First, the level of mutCALR cell surface expression is inextricably linked to expression of TPO‐R—therefore expressed at low levels, and even lower on HSCs than on megakaryocyte‐lineage cells. Whether mutCALR expression on HSCs is sufficient to allow adequate depletion of the cancer‐initiating and replenishing cells remains uncertain. This will only be evident when monitoring patients with molecular responses after treatment cessation, tracking the kinetics of mutant clone re‐expansion. Similarly, the ideal depth of molecular response is not known. MPNs emerge slowly—with the driver mutations acquired years—often several decades—prior to clinical presentation. It is therefore possible that a reduction in VAF of ~10%–20% may “re‐set the clock” sufficiently to substantially improve clinical outcomes for patients, and that very deep molecular responses may not be necessary, contrasting TKI treatment paradigms for CML. However, if the stem/progenitor cell pool is inadequately targeted, the mutant clone burden may rapidly re‐expand following cessation of therapy.

### Breaking through the immunologically hostile TME

Second, the TME in myeloid malignancies presents a hostile environment for immunotherapies. In ET and myelofibrosis, abundant bone marrow megakaryocytes harbor immunosuppressive cytokines, and studies have documented associated T cell dysfunction in mutCALR MPNs.^43^, ^44^ Whether T cell‐reliant immune therapies can overcome these obstacles in all patients and if the fibrosis will physically “shield” stem cells from targeting remains to be proven. If inhibitory antibodies can achieve meaningful disease control that lead to immune rejuvenation but not deep molecular remissions for some patients, it is possible that targeting approaches may be sequenced with monoclonal antibody treatment first, followed by an ADC, BiTE or CAR‐T cell therapy.

### Out of the frying pan, into the fire…

Although in the majority of patients, any additional somatic mutations occur in the same clone as the MPN driver mutation, they can also occur in separate clones. This can occur when the additional somatic mutation precedes the MPN driver and leaves a residual ancestral clone, or if an additional mutation occurs in a wild‐type HSC in a patient with an established mutCALR clone. Selective ablation or inhibition of the mutCALR+ clone in these cases will alter clonal competition dynamics and may spark expansion of concurrent clonal populations. An unlikely, but important theoretical possibility, is that the concurrent clones may be more deleterious than the previously dominating mutCALR clone. If expansion of concurrent malignant clones does emerge, then delineating the clonal architecture at single cell level may be required prior to treatment for patients with additional co‐mutations of concern. This concern is also a reason to encourage treatment earlier in the disease trajectory, prior to the acquisition of additional co‐mutations.

### New definitions of response and trial endpoints

Finally, if the rapid clinical and molecular responses observed in the early studies of targeted antibodies prove durable and are associated with meaningful improvements in longer‐term outcomes, a major revision of how optimal responses to therapy are defined for patients with mutCALR+ ET is required—both for implementation in the clinic, as well as for use as clinical trial and regulatory endpoints.

## CHARTING THE PATH FORWARD

MutCALR‐targeted therapies create an unprecedented opportunity to fundamentally revisit the management of MPNs. A number of mutCALR‐directed therapies are emerging, and a range of targeting modalities is likely to be required, as each offers distinct advantages and toxicities. Emerging data stimulates rethinking of current treatment and diagnostic paradigms. In a future era of mutation‐targeted therapies (also considering JAK2V617F‐ and KITD816V‐selective inhibitors), classifying MPNs based on the dominant driver mutation may be more clinically relevant than according to cell types or clinical phenotypes. This is particularly true for ET, where JAK2V617F‐ and mutCALR‐driven neoplasms exhibit such distinct pathophysiological and clinical hallmarks. Similarly, availability of tolerable and safe therapies that efficiently induce disease remission will present a compelling case for treating younger patients with so‐called “low‐risk” ET—that is, shifting treatment algorithms from focusing on thrombotic risk, to a “progression‐centric” approach (Figure 2). Even the lowest risk ET patients can suffer from a significant symptom burden that impacts work productivity, as well as an increased risk of hemorrhage, thrombosis, pregnancy complications, and premature death. This alters the economic and clinical case for treatment and shifts the paradigm of treatment goals in ET from preventing thrombo‐hemorrhagic events to inducing disease remission. As the first drugs capable of achieving substantial clonal reduction emerge in real time, the conversion should now also shift toward gathering data to inform how we refine the definition of molecular remissions, therapy targets, and, potentially, even the definition of “cure.”

## AUTHOR CONTRIBUTIONS

**Moritz Reichert**: Conceptualization; writing—original draft; writing—review and editing; visualization. **Bethan Psaila**: Conceptualization; writing—original draft; writing—review and editing; visualization; supervision.

## CONFLICT OF INTEREST STATEMENT

B.P. is a co‐founder and shareholder of Alethio Therapeutics, has received research funding from Alethio Therapeutics and Incyte, and fees for consultancy, speaking engagements, and/or advisory work from Alethio Therapeutics, Incyte, GSK, Novartis, BMS, Blueprint Medicines, Calytrix, and Damora. M.R. has no relevant disclosures.

## FUNDING

This work was supported by Senior Cancer Fellowship Funded by Cancer Research UK in partnership with Rosetrees Trust (RCCSCF‐May24/100001), Ludwig Institute for Cancer Research, and National Institute for Health Research (NIHR) Oxford Biomedical Research Centre (BRC).

## Data Availability

Data sharing is not applicable to this article as no datasets were generated or analyzed during the current study.
